# The Silent Threat of Hypokalemia after High Voltage Electrical Injuries: A Case Study and Review of the Literature

**DOI:** 10.3390/jcm13102852

**Published:** 2024-05-12

**Authors:** Maxwell B. Baker, Dhanesh D. Binda, Ala Nozari, William E. Baker

**Affiliations:** 1 Department of Anesthesiology, Boston University Chobanian & Avedisian School of Medicine, Boston, MA 02118, USA; maxb98@bu.edu (M.B.B.); ddb96@bu.edu (D.D.B.); 2 Department of Emergency Medicine, University of Vermont Robert Larner College of Medicine, Burlington, VT 05401, USA; william.e.baker@uvm.edu

**Keywords:** electrical injuries, lightning strikes, hypokalemia, metabolic disturbances, potassium channels, literature review

## Abstract

High-voltage electrical injuries, especially from lightning strikes, can cause life-threatening complications due to extreme temperature and voltage exposure. While burns and cardiac complications have been widely described, the documentation of metabolic imbalances, particularly hypokalemia, has not been as prevalent. This report focuses on a patient with profound transient hypokalemia following a lightning strike, alongside a review of three similar cases of transient hypokalemia from the literature. Our patient, a previously healthy young man, was struck by lightning and subsequently suffered transient hypokalemia with lower extremity sensory changes, which resolved after the normalization of serum potassium levels. While the exact underlying mechanisms of transient hypokalemia following high-voltage electrical injuries are unknown, we propose a multifactorial mechanism, which includes massive intracellular shifts of potassium due to elevated epinephrine levels and the prevention of potassium efflux through the electrical disruption of voltage-gated potassium channels. Our report underscores the importance of recognizing hypokalemia in patients with high-voltage electrical injuries and contributes to the understanding of the complex mechanisms involved. Further research is necessary to understand the connection between cellular changes induced by high-voltage exposure and their effects on metabolism, particularly in relation to hypokalemia.

## 1. Introduction

Each year, approximately 1000 patients die of electrical injuries in the United States (U.S.), with 400 being associated with high-voltage injuries, and 50–300 being the result of lightning strike injuries [1]. Approximately 30,000 non-fatal shocks are reported in patients, and 5% of all admissions to burn centers in the U.S. involve high-voltage electrical injuries [1]. These types of electrical injuries can lead to a myriad of unique complications, often with severe and potentially life-threatening consequences. High-voltage electrical injuries may result in damage to multiple organ systems, such as the cardiorespiratory and central nervous systems [2]. Furthermore, high-voltage electrical injuries due to lightning strikes incur an even higher mortality of up to 30%, with an increased risk of death from these cardiac or respiratory complications [2,3].

The mechanism of injury from lightning strikes is complex, as patients are exposed to extreme voltages that surpass 1,000,000 volts as well as temperatures up to 30,000 Kelvin [3,4]. Lightning injuries can occur through several mechanisms, including a direct strike, side splash, contact, ground strike, upward strike, or blast [4]. Even though a lightning strike lasts only 30 microseconds, it can still contain over 1 billion joules of energy, contributing to its high mortality rate [5]. The injuries sustained from lightning are often more severe than those from other high-voltage sources with a 76% risk of long-term sequelae in survivors [3]. While the current literature has explored cardiac complications, burns, and Lichtenberg figures, a paucity of documenting significant metabolic disturbances exists.

High-voltage electrical injuries can be associated with hyperkalemia as a result of rhabdomyolysis or tissue necrosis. When a person is struck by lightning, the intense electrical current can cause rapid and severe muscle damage. This damage can cause significant tissue swelling with a risk of developing compartment syndrome, which may require urgent fasciotomy [6]. Additionally, the breakdown of muscle tissue can release large amounts of intracellular contents, including myoglobin, creatine kinase, and potassium, into the bloodstream. The released myoglobin can accumulate in the kidneys and lead to acute kidney injury (AKI) and, in severe cases, renal failure [6]. The massive efflux of potassium from the damaged cells can exceed the kidney’s clearance limits, leading to hyperkalemia with a risk for serious cardiac effects such as dysrhythmias, cardiac arrest, and death if not promptly treated [7]. The management of hyperkalemia involves stabilizing the cellular membrane of cardiomyocytes, shifting potassium into the intracellular space, and removing excess potassium from the body [8]. Treatment may therefore involve the administration of calcium gluconate, insulin, and beta-agonists, in addition to fluid resuscitation, monitoring and management of other electrolyte imbalances, and renal replacement therapy, if necessary.

While clinicians are often vigilant regarding the potential risk of hyperkalemia following high-voltage electrical injuries, the possibility of hypokalemia is frequently overlooked. In this case, concerning the report and review of the existing literature, we examine a patient who presented with profound transient hypokalemia after being struck by lightning. We also review one additional reported case of transient hypokalemia following a lightning strike and two cases of transient hypokalemia after other types of high-voltage electrical injuries. Moreover, we explore possible mechanisms for the transient hypokalemia observed in these patients.

## 2. Case Report

A previously healthy young man presented to our emergency department after being struck by lightning. Our patient arrived at the trauma bay wet and shivering. Initial vital signs included the following: a blood pressure of 141/84 mm Hg, a heart rate of 105 beats/min (bpm), a respiratory rate of 16 breaths/minute, an oxygen saturation of 96%, and a body temperature of 97.9 °F. His hair was singed, and he had burns on his scalp, shoulders, back, and legs. Bilateral lower extremity cyanosis was observed, and both feet were cold to the touch. A partial thickness burn at the apex of the scalp was noted, which was contiguous with a series of branching linear erythematous markings extending down the patient’s back. Additional thermal injuries were present bilaterally on the patient’s hips as well as his left foot. The patient was alert and oriented to his name but confused and unable to recall the events that led up to his transport. Treatment for hypothermia consisting of a forced-air warming blanket along with warm IV fluids was initiated. The physical exam was notable for partial thickness burns of varying sizes over his torso and extremities. The patient also reported significant pain in his back and upper extremities, prompting the administration of 100 mcg of fentanyl. He further stated that he had bilateral lower extremity numbness, tingling, and weakness.

**Figure 1:**
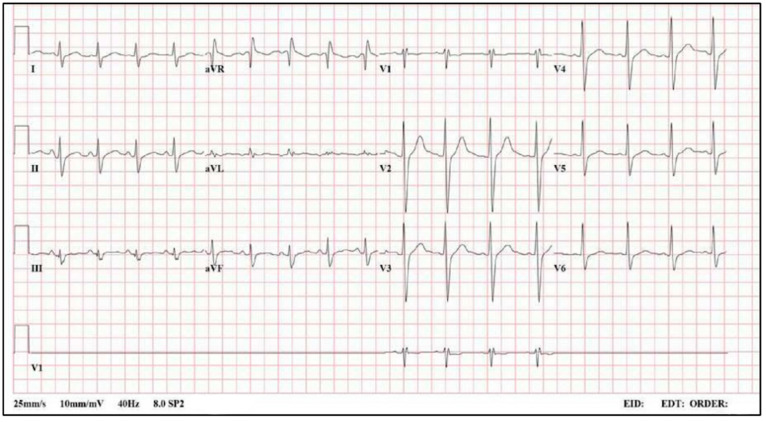
Initial ECG demonstrating a mild intraventricular conduction delay, characterized by a prolonged QTc of 461 milliseconds, merging of the T and U waves (most prominent in lead II), and distinct U wave most visible in V3. ECG, electrocardiogram.

The patient’s electrocardiogram (ECG) demonstrated a QRS duration of 110 milliseconds, a mildly prolonged QTc of 461 milliseconds (prolonged QTc defined as >450 milliseconds in male patients and >460 milliseconds in female patients) [9], and a merging of the T and U waves with the U wave being most distinctly visible in V3 (Figure 1). The initial labs revealed a serum sodium of 142 mmol/L (135–148), potassium of 2.5 mmol/L (3.5–5.3), chloride of 99 mmol/L (98–106), bicarbonate of 21.1 mmol/L (21–28), creatine kinase (CK) of 205 U/L (39–259), creatinine of 1.05 (0.8–1.3), magnesium of 2.3 mg/dL (1.6–2.6), and glucose of 102 mg/dL (65–100). An arterial blood gas (ABG) drawn on arrival demonstrated a pH of 7.21 (7.35–7.45) with a pCO_2_ of 54.2 mmHg (35–45) and a bicarbonate of 21.2 mmol/L (21–28). Fluid resuscitation and potassium repletion were initiated in the trauma room with normal saline, and the patient underwent computer tomography imaging of his head, neck, chest, abdomen, and pelvis, which showed no acute abnormalities or injuries. Thirty-two minutes after presentation to the emergency department, the patient’s serum potassium level had increased to 2.9 mmol/L, but his lower extremity numbness and paresthesia were unchanged. With continued supportive care as well as potassium and magnesium repletion, his serum potassium rose to 3.7 mmol/L six hours later, peaking at 4.5 mmol/L in the subsequent hours before stabilizing at 3.8 mmol/L on final evaluation prior to discharge. Troponin on arrival was 0.012 ng/mL, peaked at 0.301 ng/mL 10 h into the hospitalization, and was 0.015 ng/mL 46 h after the patient’s initial presentation. Approximately 70 h later, on the third day of hospital admission, the patient’s lower extremity numbness resolved, and the patient was able to ambulate, prompting subsequent discharge with topical bacitracin for wound care.

## 3. Review of the Literature

We conducted literature searches on PubMed/Medline and Google Scholar, focusing on studies published up to December 2023. We aimed to gather a range of publications, including retrospective studies, case series, and case reports, that documented instances of potassium derangements following high-voltage electrical injuries, with a particular emphasis on lightning strikes. We utilized a set of keywords relevant to our study topics (lightning strikes OR electrical injuries AND hypokalemia OR hyperkalemia OR rhabdomyolysis OR metabolic disturbances OR sensorimotor deficits OR cardiac arrhythmias OR cardiac dysrhythmias). Only English-language publications were considered.

Our search results included articles on high-voltage electrical injuries, lightning strike injuries, hypokalemia, hyperkalemia, and other metabolic derangements. We extracted and summarized the key findings and characteristics of the identified studies and cases related to hypokalemia for inclusion in Table 1 [2,10,11].

The first previously documented case details a 17-year-old woman who was struck by lightning while walking [2]. The patient arrived intubated at the trauma room with a Glasgow Coma Score of 3, a blood pressure of 100/50 mmHg, a heart rate of 110 bpm, and a temperature of 36.3 °C. The physical exam showed electrical burns on her lower extremities. Lab results indicated low serum potassium (3.1 mmol/L) and elevated CK (5783 U/L). She was diagnosed with postcardiac arrest syndrome and hypoxic-ischemic encephalopathy, leading to a targeted temperature management at 32–34 °C. The authors did not offer any insight into the cause of the observed transient hypokalemia.

Dasgupta et al. discuss a 41-year-old man whose construction vehicle came into contact with a high-voltage wire [11]. Upon arrival at the hospital, the patient was conscious but exhibited flaccid paralysis in both lower extremities. He had second to fourth-degree burns covering 40% of his body. His initial serum potassium was 1.7 mmol/L, and he received aggressive potassium repletion at 80 mmol/h. His CK levels peaked at 1058 U/L. Potassium repletion ceased after 24 h since his motor function fully returned. Investigations into his severe hypokalemia, including potential hypokalemic periodic paralysis (HPP), a rare genetic disorder that arises from a mutation in the dihydropyridine receptor, revealed no specific diagnostic conclusions [12,13]. The authors suggested that the likely cause of the hypokalemia was massive intracellular potassium shifts due to the high-voltage injury.

The third report examines a 15-year-old man who came into contact with power lines [10]. In the emergency department, he was hemodynamically stable, with mild upper and severe lower extremity weakness. An ECG showed prolonged QT intervals and T-wave abnormalities. The initial labs indicated a serum sodium of 139 mmol/L, potassium of 1.6 mmol/L, and bicarbonate of 19.7 mmol/L. The patient autonomously corrected his potassium levels. The authors concluded that the high-voltage injury likely caused muscle damage, releasing potassium into the bloodstream, which was then rapidly absorbed by healthy cells via stimulated sodium–potassium pumps (Na^+^/K^+^-ATPase). They identified three potential mechanisms for this increased Na^+^/K^+^-ATPase activity: cholinergic discharge, epinephrine signaling, and CGRP signaling [10,14].

## 4. Discussion

We described a rare case of profound transient hypokalemia and sensory-motor deficits following a lightning strike, contrary to the usual hyperkalemia caused by rhabdomyolysis or tissue necrosis after such injuries [6,15]. Our literature review found three similar cases of hypokalemia following high-voltage electrical injuries [2,10,11], with one specifically involving lightning [2]. These patients showed varying degrees of electrical burns and neurological symptoms like paralysis and extremity weakness. The exact cause of hypokalemia remains unclear, but potential factors include significant intracellular potassium shifts and muscle damage from the electrical impact [10,11].

**Figure 2:**
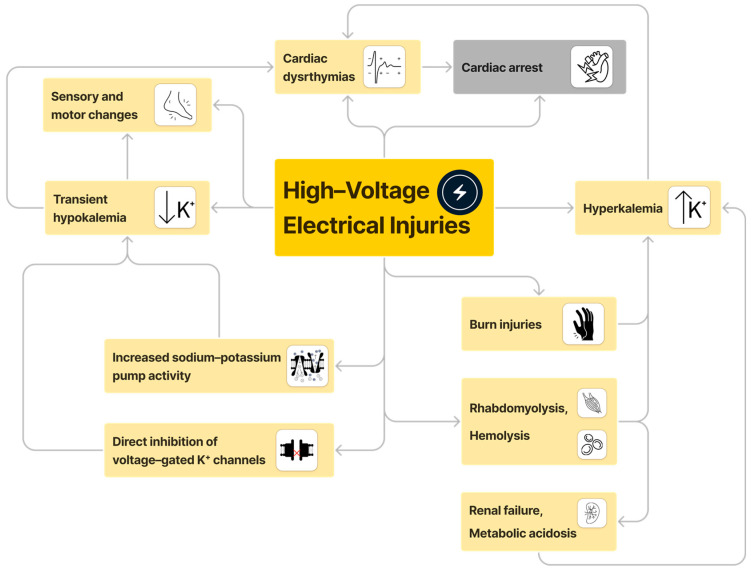
Physical, metabolic, and cellular manifestations of high-voltage electrical injuries. Na^+^, sodium, K^+^, potassium.

High-voltage electrical shock injuries can result in an array of physical, metabolic, and cellular effects (Figure 2). Significant hypokalemia is known to be associated with lower extremity sensory disturbances and paralysis [12]. Neurologic sequelae have been reported in 67% of patients who sustained high-voltage electrical shocks [16]. Sub-physiologic potassium concentrations can disrupt cellular resting potential, causing neuromuscular symptoms such as weakness, numbness, and tingling in the extremities, or even paralysis [17]. Hypokalemic paralysis can manifest as acute flaccid paralysis with areflexia and ascending paralysis, as seen in Guillain–Barré syndrome [18]. A case report of a 52-year-old man with HPP highlighted that potassium supplementation could reverse quadriparesis due to hypokalemia [19]. Direct damage to nerve tissue from high-voltage electrical injuries and secondary complications of tissue edema, ischemia, and necrosis can also induce neurological deficits [3]. Common neurologic sequelae include peripheral neuropathies, muscle weakness, sensory deficits, and autonomic dysfunction [20,21]. Electrical injuries can cause immediate and delayed neurological effects that may be underestimated initially, underscoring the need for long-term follow-up [21].

Cardiac complications from hypokalemia can be life-threatening because of the effects of low potassium levels on the cardiomyocyte’s electrical activity. Specifically, low extracellular potassium can increase the speed of diastolic depolarization, as well as conduction velocity [22]. Re-entry mechanisms or ectopic foci can lead to arrhythmias such as premature ventricular contractions and increase the risk of supraventricular arrhythmias like atrial fibrillation and flutter [23]. Hypokalemia can trigger ventricular tachycardia and fibrillation and prolong the QT interval, raising the risk of torsades de pointes [24]. For mild to moderate hypokalemia (serum levels between 3.0 and 3.5 mEq/L), oral potassium chloride supplements of 40–100 mEq per day in divided doses are recommended. Severe hypokalemia (levels below 3.0 mEq/L) requires intravenous potassium at 10–20 mEq per hour, ensuring not to exceed 40 mEq per hour or 200 mEq daily. Regular serum potassium checks every 2–4 h, continuous ECG monitoring, and vital signs assessments should be carried out [24,25]. Additionally, patients on digoxin are more vulnerable to its toxic effects when hypokalemic, requiring vigilant monitoring and potential medication adjustments [26].

The mechanism for high voltage-induced hypokalemia remains poorly understood. Following acute injuries, the immediate activation of the sympathetic nervous system leads to the increased systemic release of catecholamines, such as norepinephrine and epinephrine [27]. These neurotransmitters play a vital role in mediating the body’s counter-regulatory stress responses. For instance, circulating catecholamine levels have been correlated with the severity of injury in patients who have suffered local or multi-system trauma [28,29,30]. In myocardial infarction, studies have reported a rise in circulating epinephrine concentration up to 113 times and up to an 11-fold increase in circulating norepinephrine [31]. A similar study observed elevated plasma epinephrine levels 10 h after myocardial infarction [32]. Furthermore, a laboratory study on perfused animal adrenal glands showed that electrical stimulation induces substantial epinephrine and norepinephrine release, correlating with the stimulus’s strength and duration due to the electrotonic depolarization of medullary cells opening voltage-dependent calcium channels [33].

This catecholamine release following acute injury may contribute to hypokalemia. Studies have established a clear relationship between heightened epinephrine levels and hypokalemia [34]. Catecholamine-induced hypokalemia is thought to be primarily mediated by β2 receptors, with possible potassium influx through the stimulation of the Na^+^/K^+^-ATPase in addition to an insulin-mediated effect. A 1994 study also showed that high-voltage electrical shocks can impair voltage-gated potassium channel function. Recovery times varied, with more severe shocks leading to longer recovery periods, indicating a direct relationship between shock intensity and the extent of channel dysfunction [35]. Other mechanisms of hypokalemia may include a decreased potassium intake from poor diet or malnutrition, gastrointestinal losses from vomiting or certain diuretics, and renal salt wasting due to kidney disorders or medications [36,37,38]. Furthermore, hypomagnesemia can interfere with potassium reabsorption by affecting Na^+^/K^+^-ATPase function and hormonal regulation, exacerbating potassium loss [39]. These factors, if not addressed, can lead to severe health issues, including cardiac complications.

**Figure 3:**
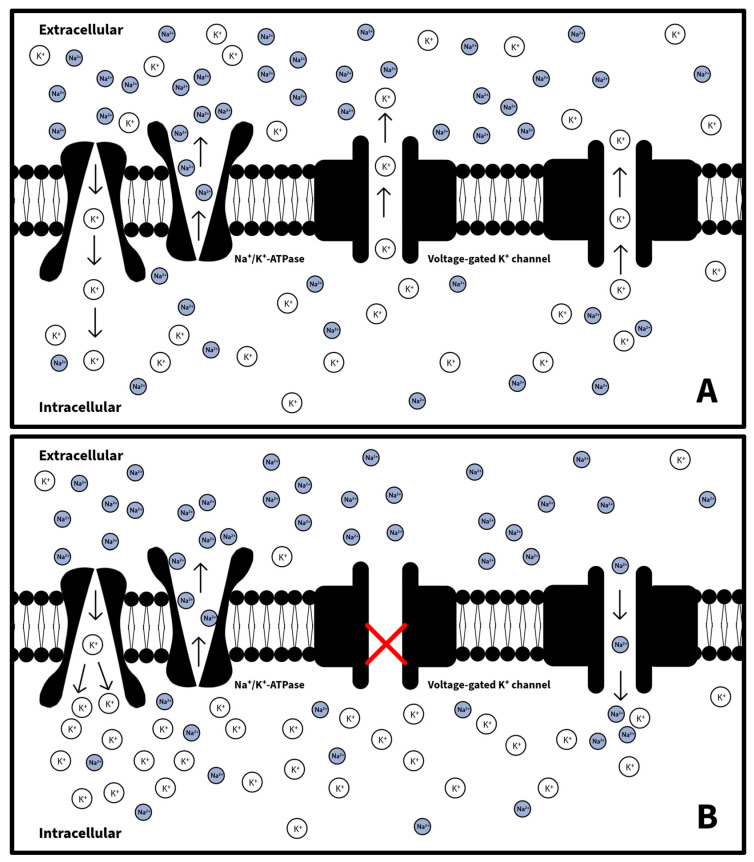
(**A**) Normal K^+^ channel and Na^+^/K^+^-ATPase function: K^+^ enters the cell via the Na^+^/K^+^-ATPase while Na^+^ exits the cell through the Na^+^/K^+^-ATPase. K^+^ exits the cell through voltage-gated K^+^ channels embedded in the cell membrane. (**B**) Following high-voltage shock, increased β2-adrenergic signaling causes heightened Na^+^/K^+^-ATPase activity, reduced K^+^ conductance through voltage-gated K^+^ channels, and reduced selectivity against Na^+^ by 50% [35]. K^+^, potassium; Na^+^/K^+^-ATPase, sodium–potassium pump; Na^+^, sodium.

Despite an initial pH of 7.21 suggesting hyperkalemia due to acidemia causing potassium to shift from cells to the extracellular space, our patient paradoxically showed transient hypokalemia. Given his good health, negative urine toxicology, and no history of significant alcohol use or the administration of potassium-lowering drugs like diuretics or beta-agonists during his admission, dietary causes or medications are unlikely explanations. We hypothesize that his hypokalemia resulted from enhanced cellular potassium uptake and reduced efflux, driven by increased Na^+^/K^+^-ATPase activity from endogenous epinephrine after the injury and possibly influenced by negative high voltage from the lightning affecting potassium channel function (Figure 3) [34]. This hypothesis aligns with similar cases of transient hypokalemia following electrical injuries, suggesting increased intracellular potassium movement as the primary mechanism. 

## 5. Limitations and Directions for Future Research

Our review has several limitations, primarily our reliance on case reports. These studies lack control groups, limiting the generalizability of our findings. Additionally, the rarity and unpredictability of high-voltage electrical injuries, especially lightning strikes, present ethical and practical challenges in assembling large, controlled cohorts. Further research should explore transient hypokalemia following high-voltage electrical injuries using retrospective cohort studies to analyze existing medical data for patterns, prospective studies in high-risk jobs to examine long-term effects on electrolyte balance, and laboratory studies on cellular mechanisms affected by electrical shocks. Establishing a national registry could support research on genetic factors influencing electrolyte imbalances post-injury. Moreover, investigating preventive and treatment strategies will be important in advancing clinical practices and improving patient outcomes related to high-voltage injuries.

## 6. Conclusions

Profound transient hypokalemia following high-voltage electrical injuries remains an underreported and understudied phenomenon, with mechanisms that are not yet fully understood. Our review of a lightning strike case and three other previously documented cases, including another lightning strike, all demonstrate profound transient hypokalemia with associated sensorimotor changes in the extremities. These cases illustrate the restoration of normal neurological function and the resolution of sensory and motor disturbances once potassium levels were corrected toward normokalemia. They underscore a notable clinical recovery despite the severity of the injuries, with rapid improvements and discharges occurring under varied circumstances. These observations support the hypothesis that exposure to extreme negative voltage electricity can significantly impact serum potassium levels through its direct effects on voltage-gated potassium channels and the Na^+^/K^+^-ATPase, potentially exacerbated by factors such as increased epinephrine signaling. However, the reliance on case reports and the absence of experimental or controlled cohort studies limit our ability to generalize these findings. Therefore, further research is necessary to explore how high-voltage electrical exposure induces these specific cellular changes and metabolic disturbances. Such studies could help to clarify the mechanisms of hypokalemia, improving both prevention and treatment protocols for those affected by high-voltage electrical injuries.

## Figures and Tables

**Table 1:** Summary of case reports detailing transient hypokalemia in patients who suffer high-voltage electrical injuries.

Authors	Mechanism of Injury	Loss of Consciousness	Initial K (mmol/L)	Extremity Findings	Cardiac Arrest	ECG Abnormalities
Baker et al.	Lightning strike	Yes	2.5	Numbness and paresthesia of bilateral lower extremities	No	Mildly Prolonged QTc, small U wave
Rotariu and Manole [2]	Lightning strike	Yes	3.1	Not reported	Yes	Not reported
Bird et al. [10]	Power line	No	1.6	Weakness of bilateral upper and lower extremities	No	Prolonged QTc, T wave abnormalities
Dasgupta et al. [11]	115 kV live wire	Yes	1.7	Flaccid paralysis of bilateral lower extremities	No	Not reported

Note: kV, kilovolts; K, potassium; ECG, electrocardiogram.

## Data Availability

Data sharing is not applicable to this article as no new data were created or analyzed in this study.
